# Bacteraemia and quick Sepsis Related Organ Failure Assessment (qSOFA) are independent risk factors for long-term mortality in very elderly patients with suspected infection: retrospective cohort study

**DOI:** 10.1186/s12879-022-07242-4

**Published:** 2022-03-13

**Authors:** Rubén Hernández-Quiles, Esperanza Merino-Lucas, Vicente Boix, Adela Fernández-Gil, Juan C. Rodríguez-Díaz, Adelina Gimeno, Beatriz Valero, Rosario Sánchez-Martínez, Jose-Manuel Ramos-Rincón

**Affiliations:** 1 Clinical Medicine Department, Miguel Hernández University, Crta. Nacional, N-332 s/n, 03550 San Joan d’Alacant Campus, Alicante, Spain; 2 Dermatology Service, General University Hospital of Alicante and Institute for Health and Biomedical Research of Alicante (ISABIAL), Av. Pintor Baeza, 12, 03010 Alicante, Spain; 3 Infectious Diseases Unit, General University Hospital of Alicante and Institute for Health and Biomedical Research of Alicante (ISABIAL), Av. Pintor Baeza, 12, 03010 Alicante, Spain; 4 Microbiology Service, General University Hospital of Alicante and Institute for Health and Biomedical Research of Alicante (ISABIAL), Av. Pintor Baeza, 12, 03010 Alicante, Spain; 5 Internal Medicine Department, General University Hospital of Alicante and Institute for Health and Biomedical Research of Alicante (ISABIAL), Av. Pintor Baeza, 12, 03010 Alicante, Spain

**Keywords:** Bacteraemia, Sepsis, Age ≥ 80, qSOFA, Survival

## Abstract

**Background:**

In older adult patients, bloodstream infections cause significant mortality. However, data on long-term prognosis in very elderly patients are scarce. This study aims to assess 1-year mortality from bacteraemia in very elderly patients.

**Methods:**

Retrospective cohort study in inpatients aged 80 years or older and suspected of having sepsis. Patients with (n = 336) and without (n = 336) confirmed bacteraemia were matched for age, sex, and date of culture, and their characteristics were compared. All-cause mortality and risk of death were assessed using the adjusted hazard ratio (aHR).

**Results:**

Compared to controls, cases showed a higher 1-year mortality (34.8% vs. 45.2%) and mortality rate (0.46 vs. 0.69 deaths per person-year). Multivariable analysis showed significant risk of 1-year mortality in patients with bacteraemia (aHR: 1.31, 95% confidence interval [CI] 1.03–1.67), quick Sepsis Related Organ Failure Assessment (qSOFA) score of 2 or more (aHR: 2.71, 95% CI 2.05–3.57), and age of 90 years or older (aHR 1.53, 95% CI 1.17–1.99).

**Conclusions:**

In elderly patients suspected of sepsis, bacteraemia is associated with a poor prognosis and higher long-term mortality. Other factors related to excess mortality were age over 90 years and a qSOFA score of 2 or more.

## Background

Bacteraemia is a major cause of mortality in high-income countries, despite advances in therapeutic strategies. Furthermore, its incidence is increasing with the proportion of very elderly people in the population [1–3], in whom infectious disease outcomes also tend to be more severe. This is due to multiple factors, including increased comorbidities and decreased functional reserve, with patients over the age of 80 years being the most likely to develop organ dysfunction [4–6].

Identifying mortality predictors in elderly patients with bacteraemia is especially important for their follow-up. Although predictors of short-term excess mortality are relatively well established, few studies provide information regarding long-term survival [7–9]. Moreover, patient-related factors, such as the level of organ dysfunction, could impact survival even more than bacteraemia, confounding results in survival studies in this population. The quick Sequential Organ Failure Assessment (qSOFA) has been useful in detecting suspected cases of sepsis associated with higher mortality, especially in elderly patients [10–12]; however, its behaviour as a long-term prognostic marker is not well known.

This study aims to compare 1-year mortality in hospitalised patients older than 80 years with and without bacteraemia and to evaluate its association with qSOFA and other clinical and laboratory data that could act as confounders.

## Methods

### Study setting

The study involved 672 patients admitted to the General University Hospital of Alicante (Spain), an acute care centre with 750 beds that cares for a population of 265,000 inhabitants. This is an observational study of paired retrospective cohorts (1:1), in which we reviewed the results of blood cultures performed on patients older than 80 years admitted to any hospital service between January 2016 and December 2017. The sample size necessary to obtain an adjusted hazard ratio (HR) different from 1 with respect to mortality between positive and negative blood culture was calculated. To do this, a pilot study was conducted with 20 people with a negative culture and 20 others with a positive culture, in which an HR of 1.3 and a mortality rate of 68% were obtained. With these values, setting the type I error at 5%, the type II error at 20%, and the exposure ratio at 1:1, at least 671 patients were needed.

### Study cohort

Patients who met at least two of the Systemic Inflammatory Response Syndrome (SIRS) criteria (fever > 38.0 °C or hypothermia < 36.0 °C, tachycardia > 90 beats/minute, tachypnea > 20 breaths/minute, leukocytosis [leukocytes > 12 × 10^9^/l] or leucopenia [< 4 × 10^9^/l]) were suspected of sepsis. At times, antibiotics were prescribed before blood culture. Blood cultures were obtained, processed, and interpreted according to the recommendations of the Spanish Society of Infectious Diseases and Clinical Microbiology (SIDCM—SEIMC) [13]. At least two bottles of haemoculture were obtained for patients (Becton Dickinson BACTEC FX blood culture system). Bacteraemia was defined as the isolation of pathogenic bacteria in the blood culture. The pathogen was identified by both traditional microbiological techniques (Gram culture and antibiogram by microdilution; Walk Away, Beckman, USA) and new techniques (MALDI-TOF; Bruker, Germany) from a blood culture. Potentially contaminating microorganisms, such as coagulase negative staphylococci, *Streptococcus* from the *viridans* group, *Propionibacterium acnes* or *Clostridium perfringens*, were included only when they were detected in at least two blood cultures [13].

**Fig. 1:**
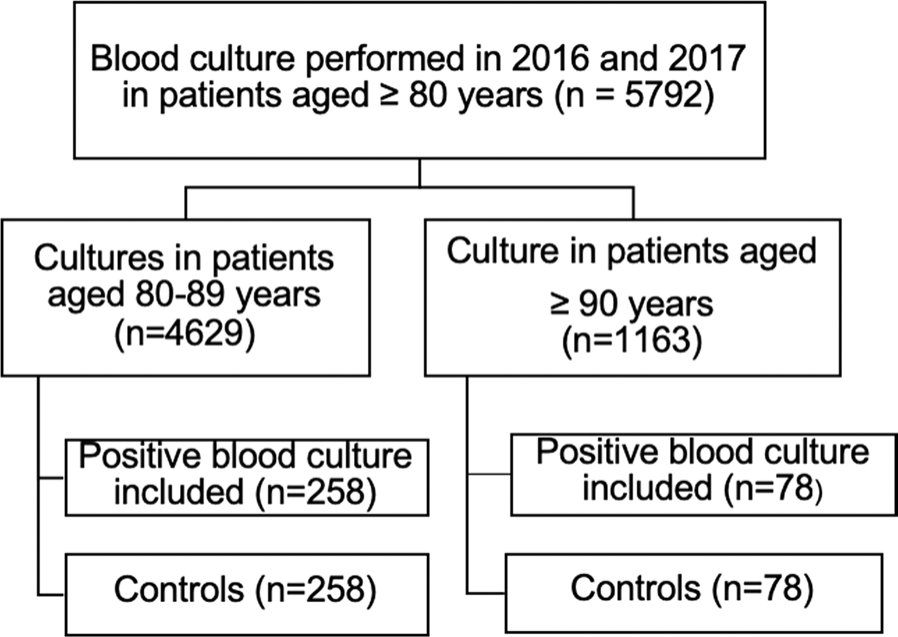
Patient selection flowchart

The cohort of patients with bacteraemia has been described previously [14]. Briefly, the exposed cohort was selected retrospectively from a database provided by the Microbiology Service at the General University Hospital of Alicante, which contained the 5792 blood cultures carried out between 2016 and 2017 in patients over 80 years of age. Patients with both community-acquired and nosocomial bacteraemia were included, while those with previous bacteraemia or unavailable clinical histories, along with cultures showing microorganisms considered to be contaminants, were excluded. In the end, 336 patients with a positive blood culture were randomly selected (Fig. 1).

All controls were selected from the same database as the cases, and they were matched for sex, age, date of blood culture, and origin of infection. If there were no equivalent controls according to these characteristics, those of the same gender and of the closest age were chosen. Controls that developed a subsequent episode of bacteraemia were included, while those with at least one previous episode were excluded. Until all the cases and controls were selected, it was unknown whether they had died at the time of the study. No data are available on how many of them had a diagnosis of infection at discharge.

### Data source

Data were collected using the unique patient identifier assigned by the Valencian Health Agency to all residents of the Valencian Community. Clinical, laboratory, and epidemiological data were obtained from each patient’s electronic health record (EHR) using the Orion Clinic v11.0 computer programme (Valencian Health Agency, Spain). The Population Information System (Generalitat Valenciana, Spain) was used to collect the date of death from any cause as well as the date of loss to follow-up. Data were stored in a Microsoft Excel v16.0 spreadsheet (Microsoft, USA).

### Variables and definitions

We collected variables registered in the EHR on the day of the blood culture. The main variable of the study was the time to event, where the event was death from any cause. Patients were classified according to their age (80–89 years vs. ≥ 90 years). Dichotomous variables for systolic blood pressure, respiratory rate, and level of consciousness were created to match the criteria of the qSOFA scale [11]: hypotension, systolic blood pressure ≤ 100 mmHg; tachypnoea, ≥ to 22 breaths per minute; and low level of consciousness, Glasgow Coma Scale < 15. Patients who met two or more qSOFA criteria were considered at high risk of organ failure. Kidney injury (KI) was defined as an estimated glomerular filtration rate (eGFR) less than 60 mL/min; hypernatremia, as a plasma sodium concentration greater than 145 mEq/L; and hyponatremia, as a concentration less than 135 mEq/L. The variables included in the study were renal function and natremia, since the relevant role of these variables as risk factors for mortality in patients with infections has been seen in other studies [15, 16]. Other variables such as lactate or albumin were not included since they are not routinely collected.

Survival was calculated from the date of the blood culture to the date of death from any cause, with a maximum follow-up time of 365 days. Using the same procedure, a 30-day survival study was also performed.

### Statistical analysis

The Kaplan–Meier method was used to perform the survival analysis and generate a graphical representation of the survival curves. Cox regression was used for the univariable and multivariable analysis; results are expressed as HR and adjusted HR (aHR), with their 95% confidence intervals (CIs). In all tests, p values of less than 0.05 were considered significant. The software used for statistical analysis was SPSS for Windows, v22.0 (IBM, USA).

### Ethics approval

The study was approved by the General University Hospital of Alicante Research Ethics Committee (CEIm PI2018/105). Analysis was performed on de-identified, aggregated patient level data, and no individual informed consent was obtained. The need for written informed consent was explicitly waived from the participants. Research was performed in accordance with the Helsinki Declaration.

## Results

### Sample description

Table 1 shows the characteristics of the sample. There were 370 women (65.1%); 516 (76.8%) were aged 80 to 89 years, while the other 156 (23.2%) were aged 90 years or more. The mean age was 86 years in patients both with and without bacteraemia. Hypotension was more frequent in cases compared to controls (24.3% vs. 16.1% p = 0.010), as was a qSOFA score of 2 or more (21.7% vs. 12.8% p = 0.003). KI was quite prevalent in both groups but more so in patients with (75%) vs. without (66.3%) bacteraemia (p=0.03). The most frequently isolated microorganism in patients with positive blood culture was *Escherichia coli* (43.2%), followed by *Enterococcus* spp. (12.4%) and *Klebsiella* spp. (10.4%). Regarding the source of bacteraemia, the urinary tract (44.3% of cases), lower respiratory tract (20.8%), and bile duct (9.3%) were the most frequent origins.

**Table 1:** Characteristics of 336 bacteraemia patients and 336 controls matched for sex, year of birth, and date of culture: Alicante, Spain, 2016–2017

	Bacteraemia patients (N = 336)	Control patients (N = 336)	p value
Gender, n (%)			
Men	151 (44.9)	151 (44.9)	
Women	185 (55.1)	185 (55.1)	
Age in years, median (IQR)	86 (82–89)	86 (82–89)	
Age group, n (%)			
80–89 years	258 (76.8)	258 (76.8)	
≥ 90 years	78 (23.2)	78 (23.2)	
Clinical situation, n (%)			
Glasgow Coma Scale < 15	98 (29.3)	85 (25.3)	0.30
Respiratory rate > 22 brpm	63 (18.8)	62 (18.5)	1.00
Systolic blood pressure < 100 mmHg	82 (24.3)	54 (16.1)	0.010*
qSOFA ≥ 2	73 (21.7)	43 (12.8)	0.003*
Biochemistry, n (%)			
Kidney injury *	253 (75.3)	222 (66.3)	0.013*
Natremia > 145 mmol/L	28 (8.3)	32 (9.5)	0.69
One-month follow-up			
Days of follow-up, median (IQR)	30 (30–30)	30 (30–30)	
Person-years at risk	23.36	24.77	
Mortality rate per person-year (95% CI)	3.08 (2.47–3.85)	1.97 (1.26–2.24)	
Deaths, n (%)	72 (21.4)	49 (14.6)	0.027*
One-year follow-up			
Days of follow-up, median (IQR)	365 (40–365)	365 (149–365)	
Person-years at risk	218.93	249.37	
Mortality rate per person-year (95% CI)	0.69 (0.58–0.80)	0.46 (0.39–0.56)	
Deaths, n (%)	152 (45.2)	117 (34.8)	0.007*
Microorganisms, n (%)			
* Escherichia coli*	145 (43)	-	
* Enterococcus* spp.	39 (12.4)	-	
* Klebsiella* spp.	35 (10.4)	-	
* Streptococcus pneumoniae*	15 (4.5)	-	
* Proteus mirabilis*	15 (4.5)	-	
Coagulase-negative staphylococci	15 (4.5)	-	
Other Enterobacteriaceae	15 (4.5)	-	
* Staphylococcus aureus*	12 (3.6)	-	
* Pseudomonas aeruginosa*	9 (2.7)	-	
Source			
Urinary tract	149 (44.3)	-	
Lower respiratory tract	70 (20.8)	-	
Biliary tract	31 (9.3)	-	
Primary bacteraemia	30 (8.9)	-	
Intra-abdominal	19 (5.7)	-	
Skin and soft tissue	15 (4.5)	-	
Catheter-related infection	16 (4.8)	-	
Other	6 (1.8)	-	

*Kidney injury is estimated glomerular filtration rate < 60 mL/min IQR: interquartile range. qSOFA: quick Systematic Organ Failure Assessment. CI: confidence interval. brpm: breaths per minute. eGFR: estimated glomerular filtration rate

### Mortality study

Table 2 shows 1-month and 1-year mortality. At 1 year, 269 of the 672 patients (40%) had died. In patients with bacteraemia, mortality was 45%, with an incidence rate of 0.69 deaths per person-year. In the case of patients with negative blood culture, it was 35% and the incidence rate was 0.46 per person-year. The observed mortality was also higher in patients aged over 90 years (51.3% vs. 36.6% p < 0.001), patients with a qSOFA ≥ 2 (68.1% vs. 34.2% p < 0.001), and with hypernatremia (56.7% vs. 38.4% p = 0.009).

**Table 2:** One-month and 1-year mortality according to exposure factor, sex, age group, quick Sequential Organ Failure Assessment (qSOFA), renal function and natremia

	One-month mortality			One-year mortality		
	Deaths/total at risk (%)	95% CI	p value	Deaths/total at risk (%)	95% CI	p value
Exposure						
Bacteraemia	72/336 (21.4)	(0.17–0.26)	0.027*	152/336 (45.2)	(0.40–0.50)	0.007*
Controls	49/336 (14.6)	(0.11–0.19)		117/336 (34.8)	(0.29–0.40)	
Gender						
Men	62/302 (20.5)	(0.16–0.26)	0.15	131/302 (43.4)	(0.44–0.60)	0.13
Women	59/370 (15.9)	(0.13–0.20)		138/370 (37.3)	(0.33–0.41)	
Age group						
80–89 years	82/516 (15.9)	(0.13–0.20)	0.013*	189/516 (36.6)	(0.33–0.43)	< 0.001*
≥ 90 years	39/156 (25.0)	(0.19–0.32)		80/156 (51.3)	(0.38–0.49)	
qSOFA						
≥ 2	53/116 (45.7)	(0.37–0.56)	< 0.001*	79/116 (68.1)	(0.60–0.77)	< 0.001*
< 2	68/556 (12.2)	(0.10–0.15)		190/556 (34.2)	(0.30–0.38)	
Kidney injury*						
Yes	92/475 (19.4)	(0.15–0.23)	0.201	198/475 (41.6)	(0.37–0.46)	0.228
No	29/196 (14.8)	(0.11–0.21)		71/196 (36.2)	(0.30–0.44)	
Natremia						
> 145 mmol/L	24/60 (40.0)	(0.29–0.54)	< 0.001*	34/60 (56.7)	(0.45–0.70)	0.009*
≤ 145 mmol/L	97/612 (15.8)	(0.13–0.19)		235/612 (38.4)	(0.35–0.42)	

*Kidney injury is estimated glomerular filtration rate < 60 mL/min CI: confidence interval. eGFR: estimated glomerular filtration rate

### One-year survival

**Fig. 2:**
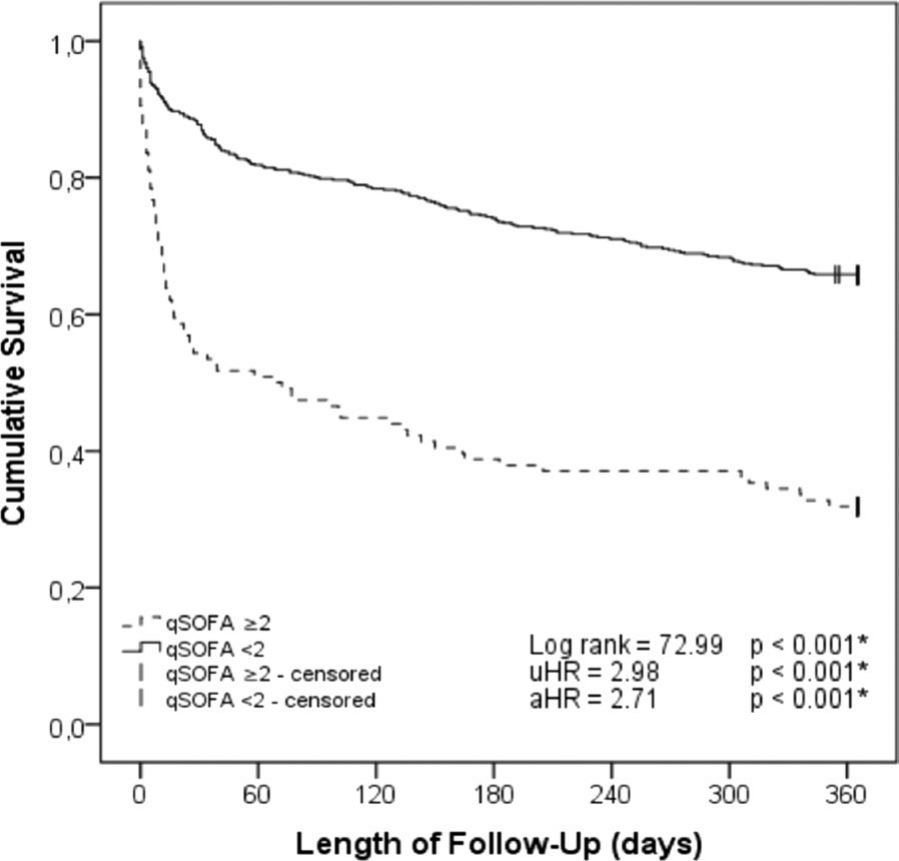
One-year survival in patients with bacteraemia vs. controls

The survival functions for both cohorts, obtained by the Kaplan–Meyer method, are represented in Fig. 2 (log-rank p = 0.004). Bacteraemia was significantly associated with mortality (HR = 1.42, log-rank p = 0.004) in the univariable analysis (see Table 3), as was age over 90 years (HR = 1.56, p = 0.001) and hypernatremia (HR = 1.94, p < 0.001). The association of the greatest magnitude was a qSOFA score of 2 or more (HR = 2.98, p < 0.001).

**Table 3:** One-month and 1-year mortality rate, univariate and multivariate analyses of the risk of death (Hazard Ratio) depending on presence of bacteraemia, age group, sex, qSOFA score, renal function and natremia

Variable	Follow-up length, days^a^	Mortality Rate^b^	95% CI	Crude analysis			Multivariable analysis		
				HR	95% CI	p value	Adjusted HR	95% CI	p value
One-month follow-up									
Exposure									
Bacteraemia	23.36	3.08	(2.47–3.85)	1.53	(1.07–2.20)	0.021*	1.30	(0.90–1.88)	0.16
Controls	24.77	1.97	(1.26–2.24)	Ref			Ref		
Age group									
≥ 90 years	10.50	3.71	(2.59–4.88)	1.66	(1.14–2.44)	0.009*	1.49	(1.01–2.19)	0.044*
80–89 years	37.63	2.17	(1.71–2.65)	Ref			Ref		
Gender									
Men	21.35	2.90	(2.18–3.63)	1.29	(0.91–1.85)	0.15			
Women	26.77	2.20	(1.63–2.74)	Ref					
qSOFA									
≥ 2	6.35	8.34	(6.10–10.59)	4.60	(3.21–6.60)	< 0.001*	3.87	(2.64–5.78)	< 0.001*
< 2	41.78	1.62	(1.24–2.01)	Ref			Ref		
Kidney injury*									
Yes	33.56	2.74	(2.18–3.30)	1.36	(9.00–2.07)	0.096			
No	14.57	1.99	(1.27–2.71)	Ref					
Sodium									
> 145 mmol/L	3.38	7.08	(4.25–9.92)	2.97	(1.90–4.65)	< 0.001*	1.76	(1.09–2.83)	0.019*
≤ 145 mmol/L	44.74	2.16	(1.74–2.60)	Ref			Ref		
One-year follow-up									
Exposure									
Bacteraemia	218.93	0.69	(0.58–0.80)	1.42	(1.12–1.81)	0.004*	1.31	(1.03–1.67)	0.029*
Controls	249.37	0.46	(0.39–0.56)	Ref			Ref		
Age group									
≥ 90 years	96.23	0.83	(0.65–1.01)	1.56	(1.20–2.03)	0.001*	1.53	(1.17–1.99)	0.002*
80–89 years	372.04	0.51	(0.44–0.58)	Ref			Ref		
Gender									
Men	203.24	0.65	(0.53–0.76)	1.21	(0.95–1.53)	0.12			
Women	265.02	0.52	(0.43–0.61)	Ref					
qSOFA									
≥ 2	49.48	1.60	(1.24–1.95)	2.98	(2.98–3.88)	< 0.001*	2.71	(2.05–3.57)	< 0.001*
< 2	418.78	0.81	(0.71–0.94)	Ref			Ref		
Kidney injury*									
Yes	327.64	1.27	(1.10–1.46)	1.20	(0.91–1.57)	0.19			
No	140.62	2.58	(2.04–3.25)	Ref					
Sodium									
> 145 mmol/L	29.81	1.14	(0.76–1.52)	1.94	(1.35–2.78)	< 0.001*	1.37	(0.94–1.99)	0.10
≤ 145 mmol/L	438.45	0.54	(0.47–0.60)	Ref			Ref		

*CI* confidence interval

*Kidney injury is estimated glomerular filtration rate < 60 mL/min HR: hazard Ratio. qSOFA: quick Systematic Organ Failure Assessment. eGFR: estimated glomerular filtration rate. ^a^Measured in person years. ^b^Rate per 1 person-year

**Fig. 3:**
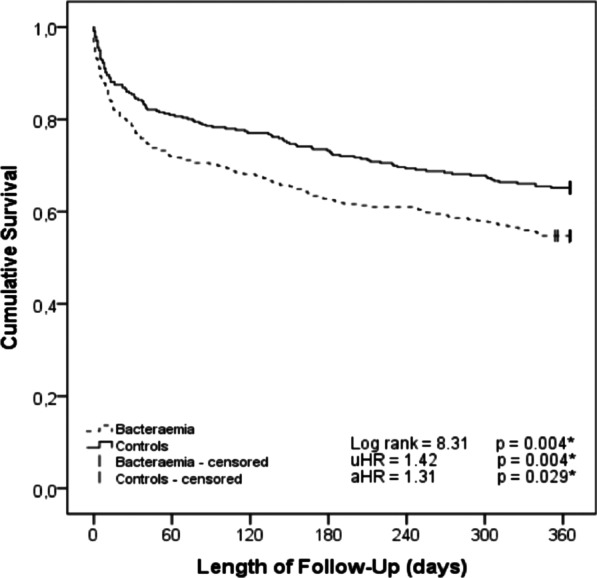
One-year survival in patients with qSOFA scores of ≥ 2 vs. < 2

In the multivariable analysis, bacteraemia remained an independent risk factor for 1-year mortality (aHR 1.31, p = 0.029). Patients over the age of 90 years (aHR 1.53, p = 0.002) and with a qSOFA score ≥ 2 (aHR = 2.71 p < 0.001) also carried a higher risk. After adjusting the data for potential confounders, no statistically significant differences were found for hypernatremia. Figure 3 shows the survival curve in patients classified according to their qSOFA score.

## Discussion

The results of the present study show how the alteration of certain variables is associated with a worse prognosis in elderly patients with suspected sepsis, although its impact on short- and long-term mortality differs. Specifically, we found that the risk of death in the first year in patients with a positive blood culture is 31% higher than those with a negative blood culture. Moreover, patients over the age of 90 have about 50% higher risk than those aged 80–89 years; and a qSOFA score of 2 or more was associated with a 171% increase in 1-year mortality.

Bacteraemia has a notable social and health impact due to its frequency and associated mortality, especially in the elderly population. Thus, the identification of prognostic factors when faced with clinical suspicion acquires special relevance in these patients. In this study, we hypothesised that a positive blood culture could have less prognostic impact than other variables such as age, clinical severity, or other laboratory findings. However, we found that bacteraemia is associated with a higher mortality in both the short and long term in very elderly patients. One-month mortality in patients with bacteraemia was 21.4%, a similar figure to that obtained in other studies, such as the one carried out by Søgaard et al. in 2008 [9]. To the best of our knowledge, ours is the first study comparing long-term mortality specifically in elderly patients using hospital controls; other authors, such as Nielsen et al. [17], reported similar mortality in this subgroup in 2015 (53% vs. 45% in this study), although with risk ratios that were 40 times higher (compared to our 31%) since they used healthy population controls.

Age has been widely described in the literature as a poor prognostic factor in patients with bacteraemia. Different authors have found 21–35% higher short- and long-term mortality in patients older than 80 years with respect to the rest of the geriatric population [5, 18–20], and Nielsen et al. [17] suggested that age may be a more important risk factor for mortality than bacteraemia itself, since the higher risks of mortality (compared to controls) were described in young patients without comorbidities. There are no studies comparing the risk in patients aged 80–89 years vs. 90 years or older, but our data show a 50% higher risk of death in the older group, both at 30 days and 1 year. This value is strikingly higher than the 35% described for those over 80 years of age, probably due to the greater number of comorbidities and higher rates of institutionalisation, as well as the lower functional reserve of the oldest patients [6].

The results suggest a higher frequency of qSOFA scores over 2 in patients with a positive blood culture, as well as a strong association with mortality, with a three-fold greater risk of death in the first month and a two-fold higher risk in the first year. Although our figures seem to indicate that qSOFA is the best predictor of mortality among the studied variables, literature on the prognostic validity of the scale is controversial. In this study, 30-day mortality was 45.7%, higher than in other studies in the elderly population, where figures ranged from 24 to 33%; In those studies, the qSOFA yielded a lower prognostic validity [12, 21, 22]. A recent systematic review [23] attributed the variability of results to the different populations studied and to differences in the interpretation of the scale’s variables, especially “altered mental state”. For this reason, a standardised definition is necessary for future studies, especially in patients with dementia and other underlying neurological disorders, very common in elderly patients.

Moreover, diagnosing bloodstream infections and sepsis in the elderly is challenging, as the expected clinical presentation of infections is often lacking or altered due to impaired ability to communicate, underlying conditions, and previous alterations in laboratory markers. Sometimes, doctors do not suspect sepsis until it is fully established.

Studies on the long-term prognosis following bacteraemia infection are scarce, and barely any data are available in elderly patients. This study provides useful information on the prognosis of bacteraemia in very elderly patients.

However, it has some limitations. First, its retrospective nature entails an inherent risk of selection or misclassification bias. Secondly, several variables were not considered, such as lactate, albumin, comorbidities, immunity status, prescribed antibiotic treatment, cause of death, or quality of life after bacteraemia. Third, the EHR had no information on frailty status, a physiological condition characterised by a decreased reserve to stressors [24]. Reliable, easy-to-use, and validated instruments are available, such as the Clinical Frailty Scale [25–29], and this scale has been shown to correlate with outcomes for several pathologies in multicentre studies [25–29]. Unfortunately, this variable was not available to us. Fourthly, delirium is associated with higher mortality, and it is one of the most consequential geriatric syndromes, especially in septic patients, but it was not considered in our study despite its potential influence on mortality. Fifthly, appropriate empirical antibiotic treatment and implementation of resuscitation bundles are a strategy for preventing mortality due to bloodstream infection, but in our study this was not recorded. Finally, it is an observational study from a single centre, which could limit the generalisability of our results.

## Conclusions

Based on our data, bacteraemia is associated with a poor prognosis and higher long-term mortality in elderly (≥ 80 years) patients. This infection remains a serious and life-threatening clinical entity. In elderly patients, optimising long-term outcomes in survivors hinges on high suspicion, early diagnosis and treatment of bloodstream infections and sepsis, and awareness of the need for multidisciplinary care, preferably involving geriatricians.

Bacteraemia should not be considered a benign process, so it is necessary to adopt measures to prevent death in the near term as well as to minimise the functional damage that can condition mortality later on. Other factors related to excess mortality were age over 90 years and a qSOFA score of 2 or more. On these last two points, little information is available, so future studies are needed.

It is important to design a multicentre prospective study on sepsis in the elderly population, including an appropriate antibiotic and implementation of resuscitation bundles, but also to optimise care of these patients, addressing risk factors like delirium and providing the elderly with the best possible care to improve short- and long-term outcomes.

## Data Availability

All data generated or analysed during this study are included in this published article.

## Abbreviations

CEIm: General University Hospital of Alicante Research Ethics Committee
SIDCM–SEIMC: Spanish Society of Infectious Diseases and Clinical Microbiology
eGFR: Estimated glomerular filtration rate
EHR: Electronic health record
HR: Hazard ratio
aHR: Adjusted hazard ratio
CI: Confidence interval
qSOFA: Quick Sequential Organ Failure Assessment
Brpm: Breaths per minute
